# Tectal Plate Glioma Presenting With Intratumoral Hemorrhage

**DOI:** 10.7759/cureus.80813

**Published:** 2025-03-19

**Authors:** Justin N Passman, Heshwin Singh, Bayan Razzaq, Charles B Mikell, David A Chesler

**Affiliations:** 1 Neurological Surgery, Renaissance School of Medicine at Stony Brook University, Stony Brook, USA; 2 Neurosurgery, Stony Brook University, Stony Brook, USA

**Keywords:** case report, intracranial hemorrhage, midbrain tumor, midbrain tumor hemorrhage, tectal glioma

## Abstract

Tectal gliomas are rare brainstem tumors. These tumors typically cause obstructive hydrocephalus due to mass effect on the cerebral aqueduct; however, intratumoral hemorrhage is exceedingly rare, with only one previously documented case to our knowledge. Patients typically present with symptoms of hydrocephalus, including headaches, nausea, and visual disturbances. We report the case of a 43-year-old man with a known tectal plate glioma who presented with acute obstructive hydrocephalus secondary to intratumoral hemorrhage. Following the patient’s rapid neurological decline, computed tomography (CT) and magnetic resonance imaging (MRI) confirmed the diagnosis. The patient underwent an endoscopic third ventriculocisternostomy (ETV) for cerebrospinal fluid diversion, along with an endoscopic biopsy of the tectal mass. The patient’s postoperative course was favorable, with gradual resolution of symptoms, including diplopia and headaches. A follow-up MRI revealed reduced tumor size and stable ventriculomegaly. Histopathological analysis suggested the tumor to be of glial origin and low-grade in nature, based on its contrast enhancement on MRI and the patient’s clinical trajectory. This case illustrates a rare presentation of intratumoral hemorrhage in tectal gliomas, emphasizing the need for heightened clinical suspicion in such cases. ETV remains an effective treatment for obstructive hydrocephalus, though the potential for hemorrhagic complications warrants close monitoring.

## Introduction

Tectal plate gliomas are a rare class of brainstem tumors that encompass approximately 8% of brainstem tumors and 2% of gliomas overall in adults [1]. These tumors are more common in children, but in adults, they typically present in the mid-to-late 4th decade and are more common in men [2]. Compared to other brainstem tumors, such as classic diffuse pontine glioma, low-grade gliomas of the midbrain tectum are often contrast-enhancing on neuroimaging and carry an indolent course [3]. Presenting symptoms often include headache secondary to hydrocephalus or contralateral cranial nerve deficits from mass effect [1-3]. Tectal plate gliomas often originate near the cerebral aqueduct, leading to obstructive hydrocephalus [3].

Although mixed appearances are common, tectal plate gliomas typically have an astrocytic or oligodendrocytic predominance [3]. Most tectal plate gliomas are WHO Grade I and have a favorable prognosis with watchful waiting and endoscopic third ventriculocisternostomy (ETV) as needed [3]. However, glioblastoma multiforme (WHO Grade IV) in the brainstem has also been documented, which has a very poor prognosis [3]. At the time of ETV, a lesion biopsy can be performed for microscopic and genetic characterization [3]. Surgical resection and chemoradiation are typically reserved for benign lesions but are usually employed for higher-grade lesions [3].

In this case report, we present the case of a 43-year-old man with a history of a known tectal plate glioma that presented with severe hydrocephalus secondary to intratumoral hemorrhage. To our knowledge, this is only the second documented case, and the first in the last 17 years, of the presentation of tectal plate glioma secondary to intratumoral hemorrhage and not obstructive hydrocephalus secondary to mass effect.

## Case presentation

**Figure 1:**
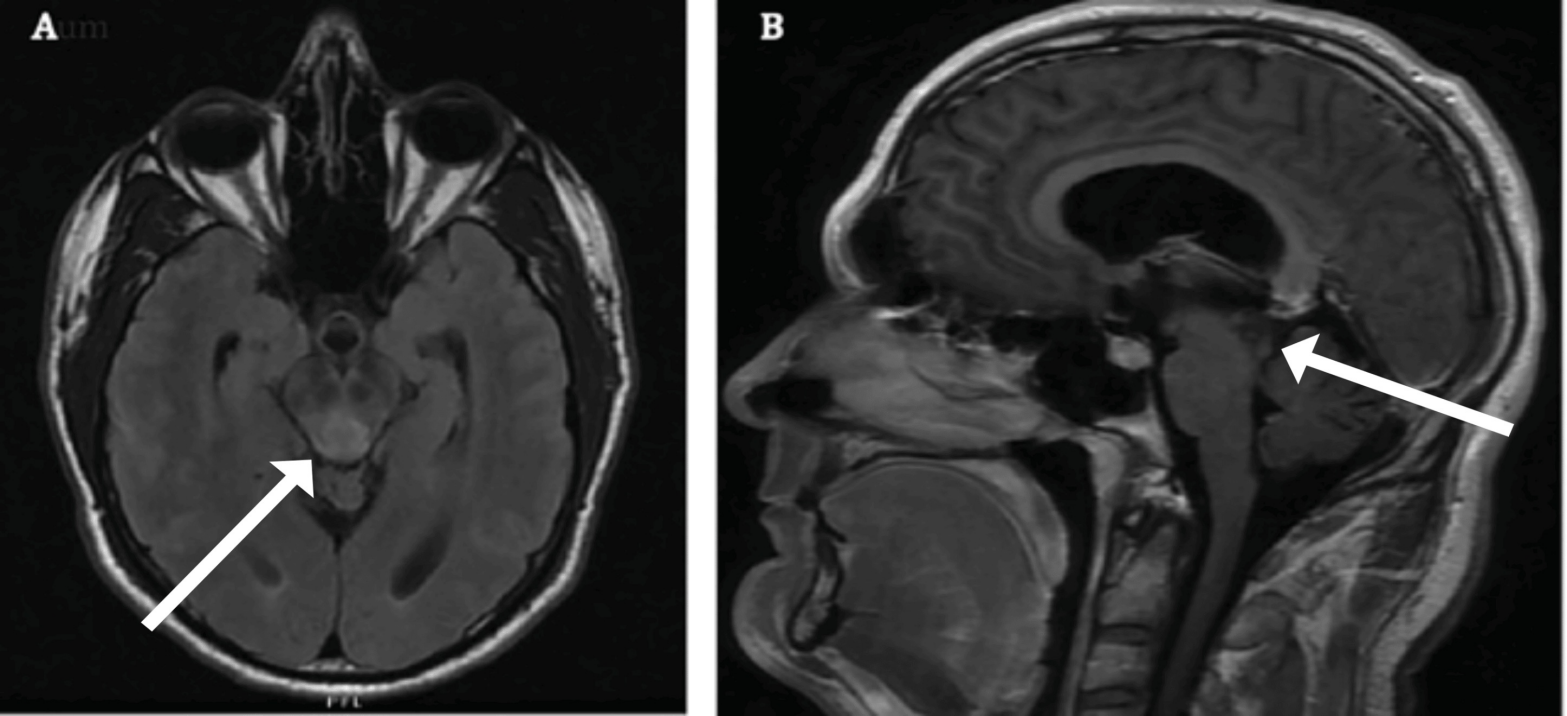
(A) Axial T2-weighted MRI of the brain with FLAIR at initial presentation to the neurosurgical oncology clinic. (B) Sagittal T1-weighted MRI of the brain post-contrast. Sagittal T1-weighted and axial T2-weighted FLAIR MRI reveal a T2 hyperintense lesion centered in the midbrain tectum with extension into the right thalamus, producing a mild mass effect on the third ventricle and compressing the aqueduct of Sylvius, leading to mild hydrocephalus. There is no abnormal restricted diffusion, midline shift, intracranial hemorrhage, or extra-axial collection. Major arteries in the circle of Willis show normal flow voids, and no significant abnormalities are noted in the pituitary gland, orbits, or paranasal sinuses. This lesion likely represents a glioma, and further evaluation with contrast-enhanced imaging is advised for better characterization. FLAIR: fluid-attenuated inversion recovery.

This patient was a 43-year-old man with no past medical history who presented to the neurosurgical oncology clinic two months after being assaulted and sustaining a head injury. He had no loss of consciousness and underwent minor laceration repair. An MRI showed a non-enhancing lesion at the opening of the cerebral aqueduct but no other signs of injury or hemorrhage (Figure 1 A and B). He was then referred to our neurosurgical oncology clinic.

Upon initial evaluation at our clinic, the patient had no complaints and denied headache, vision changes, language disturbances, gait disturbance, incontinence, nausea, vomiting, fevers, weight changes, or any other symptoms. He was able to continue his exceedingly active lifestyle and had a normal physical exam. The patient’s hydrocephalus was mild and asymptomatic. The clinical determination was that the patient likely had a WHO Grade 1 tectal glioma. The plan was to follow the patient with serial MRI and clinical exams. At a three-month follow-up, the patient’s history, physical exam, and MRI findings remained unchanged. The patient continued to report feeling well. As the patient’s status remained stable, the shared decision was to follow up in one year with an MRI and exam, sooner if needed, for any changes in symptoms or concerns.

**Figure 2:**
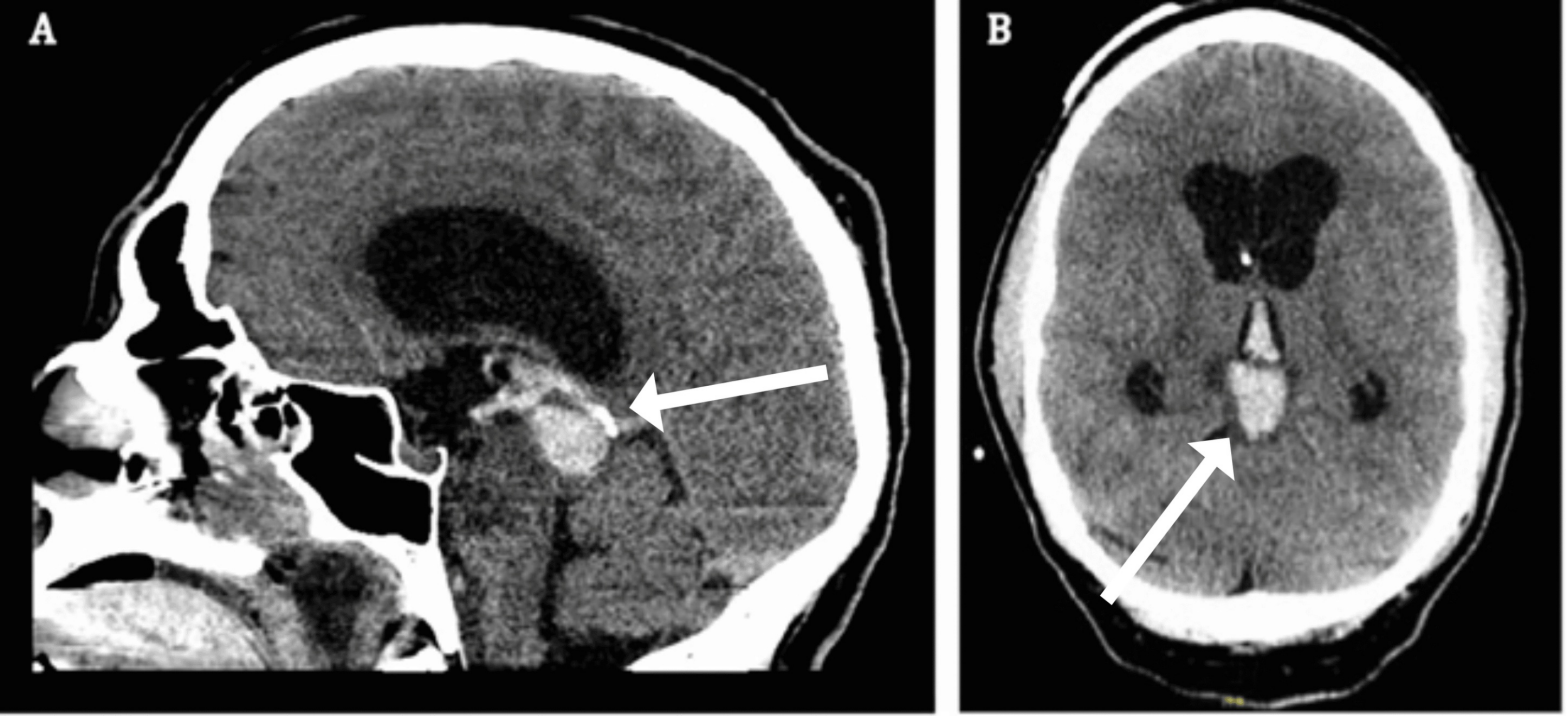
(A) Sagittal non-contrast CT head at ED presentation for decreased level of consciousness. (B) Axial non-contrast CT head at ED presentation for decreased level of consciousness. Acute intracranial hemorrhage appears centered in the tectum, measuring up to 2.1 × 1.6 × 1.7 cm, extending into the third ventricle and dependent on bilateral lateral ventricles. A right frontal approach ventricular catheter terminating in the midline frontal horn of the right lateral ventricle is seen. Mild diffuse ventriculomegaly is compatible with hydrocephalus. Effacement of convexity sulci may reflect diffuse cerebral edema. ED: emergency department.

Six months following his initial presentation to our clinic, the patient reported severe headaches to his wife in the evening. Later that evening, the patient had an episode of witnessed vomiting and became obtunded. The patient was brought to a community hospital, and the CT head was significant for pineal intracranial hemorrhage with acute-onset hydrocephalus secondary to an intratumoral hemorrhage of his known tectal glioma, with the extension of the hemorrhage to the third ventricle (Figure 2 A and B). An extraventricular drain was placed at 15 cm H₂O, with an opening intracranial pressure of 12 mmHg. The patient was intubated, given Ativan, and loaded with Keppra for concern of seizure activity. The patient was transferred from the ICU at the community hospital to our institution’s ICU for a higher level of care. The patient was taken to the operating room with our on-call neurosurgery team for definitive treatment of his hydrocephalus as well as an attempted endoscopic biopsy of the mass. Endoscopic third ventriculocisternostomy through a right frontal approach and endoscopic biopsy of the tectal mass were performed. Cerebrospinal fluid samples were obtained for standard studies, microbiology, and cytopathology.

Postoperative course

**Figure 3:**
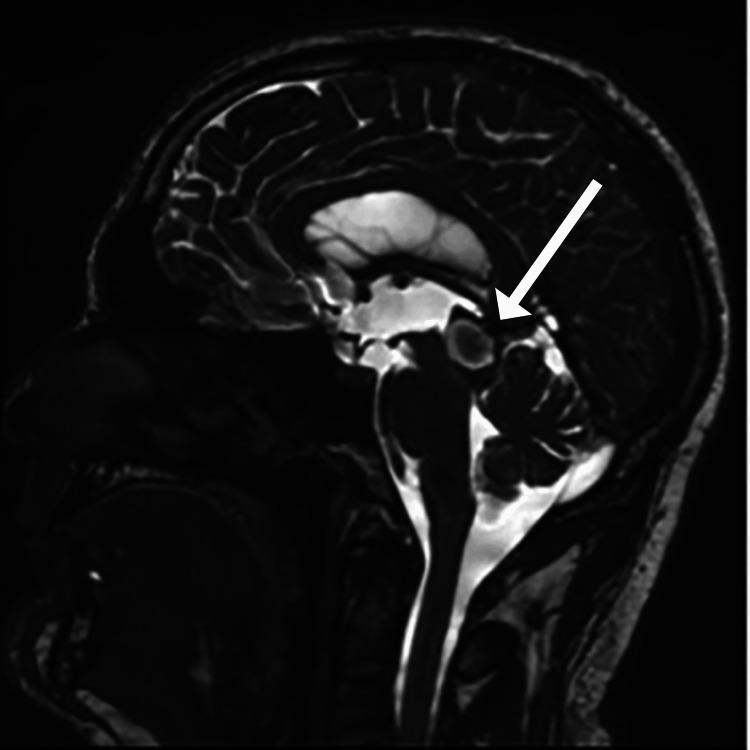
T1-weighted MRI of the brain with FLAIR showing an evolving hemorrhage at the midbrain/tectum, measuring approximately 1.9 × 1.5 × 2.2 cm (axial × sagittal × craniocaudal), and in the lateral and third ventricles, with corresponding T1 hyperintensity. Interval increase in patchy foci of restricted diffusion with FLAIR hyperintensity at the bilateral frontal vertices suggests possible small thromboembolic infarctions. A stable right frontal ventriculostomy catheter is present. FLAIR: fluid-attenuated inversion recovery.

The patient was regularly followed for the next 1.5 years, during which he continued to experience diplopia, monitored by neuro-ophthalmology. Over time, the diplopia gradually resolved, and the patient reported significant symptomatic relief from preoperative headaches and nausea, stating he felt generally well. A one-month postoperative MRI of the brain, with and without intravenous contrast, revealed postoperative changes consistent with a right frontal burr hole and a gliotic tract extending through the frontal lobe to the frontal horn of the lateral ventricle (Figure 3). There was stable, mild ventriculomegaly in the lateral and third ventricles, with a normal appearance of the fourth ventricle. A hemorrhagic mass expanding the tectum and obstructing the aqueduct of Sylvius was observed, with a slight decrease in size from prior imaging. CSF flow studies confirmed brisk flow through the cisternostomy defect and an absence of flow through the aqueduct, indicating treated obstructive hydrocephalus and patency of the ventriculocisternostomy. At the follow-up visit, the patient reported continued improvement in his diplopia, with neuro-ophthalmology noting further progress. He denied any symptoms of nausea, vomiting, dizziness, or headaches. On examination, he was awake, alert, and oriented. His cranial nerves were intact bilaterally, with mild but improved restriction in upgaze. No lid lag was observed, and the previously noted disconjugate gaze had resolved. His exam was otherwise normal.

**Figure 4:**
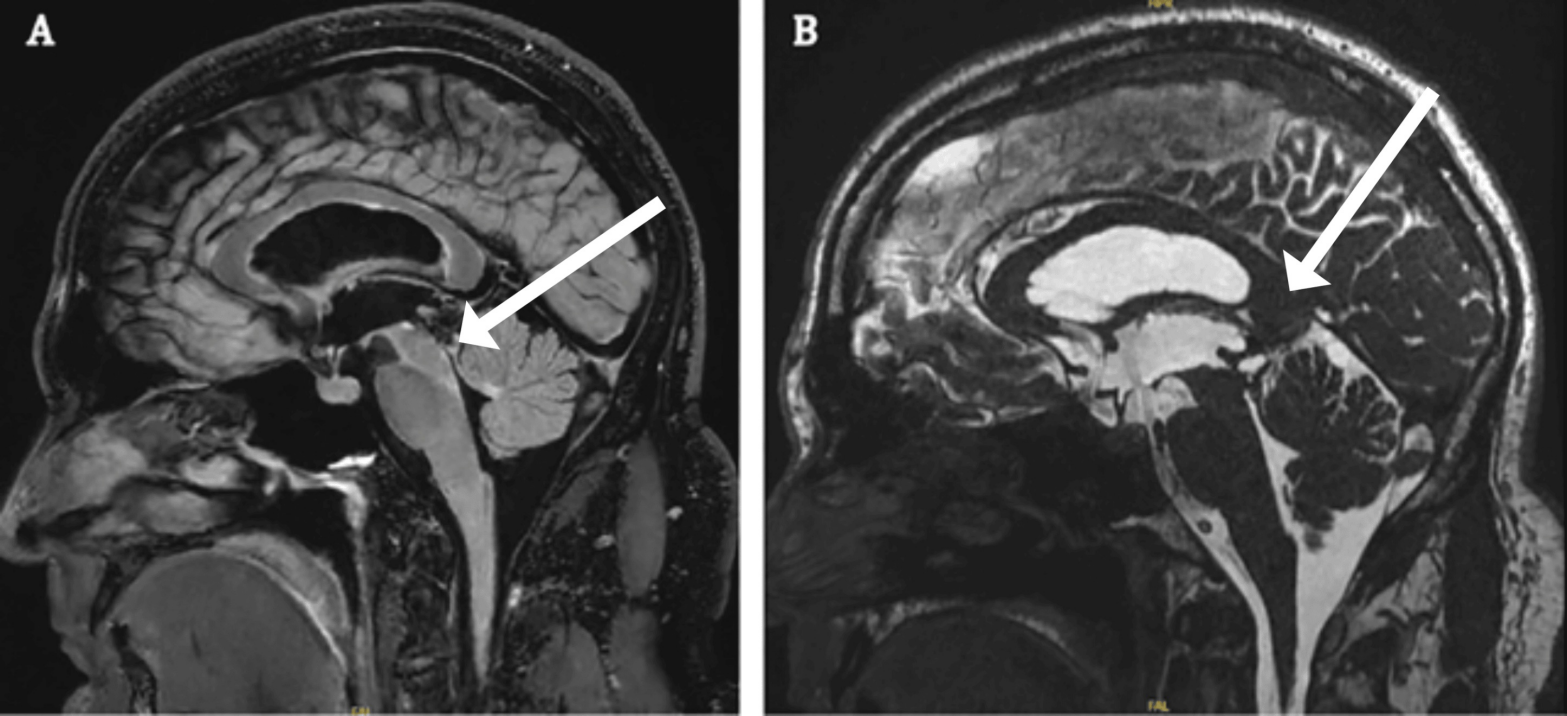
(A) Sagittal T2-weighted MRI of the brain with FLAIR 1.5 years post-ETV. (B) Sagittal MRI of the brain with 3D FIESTA, 1.5 years post-ETV. A stable T1 hypointense, T2 hyperintense, and heterogeneous hemorrhagic lesion is present in the tectum with associated FLAIR hyperintensity in the bilateral thalami, more prominent on the right, where a 0.9 × 0.9 × 0.7 cm lesion shows susceptibility consistent with chronic blood products. There is no CSF flow through the cerebral aqueduct; however, a patent third ventriculostomy demonstrates expected CSF flow dynamics. A mild interval increase in lateral and third ventricular size is noted, with no evidence of acute hemorrhage. CSF: cerebrospinal fluid, ETV: endoscopic third ventriculostomy, FLAIR: fluid-attenuated inversion recovery, FIESTA: fast imaging employing steady-state acquisition, MRI: magnetic resonance imaging.

A 1.5-year postoperative MRI of the brain confirmed postoperative changes consistent with the right frontal endoscopic third ventriculocisternostomy, with CSF flow studies showing continued patency (Figure 4A and 4B). The tectal lesion remained heterogeneously dark on T1 and bright on T2, with adjacent signal abnormalities extending into the bilateral thalami. No conclusive postcontrast enhancement of the lesion was observed. Ventricular size remained normal, and there was no evidence of acute infarct or additional mass lesions. Multiple biopsy sections were prepared, with tissue stained at four depths using H&E. The findings were suggestive of, but not definitive for, a glial neoplasm, given the presence of cells with round nuclei and eosinophilic processes in a myxoid background [4].

## Discussion

Observations

Gliomas of the tectal plate are rare brainstem tumors that typically present with symptoms of obstructive hydrocephalus secondary to mass effect. However, presentation with intratumoral hemorrhage is exceedingly rare, with only one prior documented case to our knowledge. In this case report, we present a 43-year-old man who developed severe obstructive hydrocephalus secondary to intratumoral hemorrhage of a known tectal plate glioma. Hemorrhage of glioblastoma multiforme in the brainstem has been documented, resulting in the death of the reported patient [5]. In this case, however, the fatal hemorrhage occurred after the placement of the ETV, likely due to injury to the tumor [5].

Intratumoral hemorrhage within tectal gliomas is an uncommon but potentially devastating complication, especially when compounded by obstructive hydrocephalus [6]. Tectal gliomas, which are predominantly low-grade, usually lead to hydrocephalus due to mass effect on the cerebral aqueduct, blocking cerebrospinal fluid (CSF) flow [7]. Hemorrhagic events within these tumors, though rare, can worsen intracranial pressure, making the clinical situation more urgent [6].

Biopsy is rarely performed in gliomas of the tectum due to concerns about causing hemorrhage and the increased rates of morbidity and mortality associated with biopsy [8]. Additionally, tectal plate gliomas are traditionally diagnosed via imaging and are considered low-grade based on survival trajectory and contrast enhancement on MRI [3, 9, 10]. Given that this patient continues to live and has returned to full activity, there is a fair degree of certainty that the pathology is consistent with a low-grade glioma, as is most common. If this were a high-grade glioma, the patient likely would have shown radiographic and/or clinical progression by this time.

In our case, the patient developed acute obstructive hydrocephalus due to intratumoral hemorrhage within a known tectal glioma. Although this is an extremely rare presentation, successful treatment with ETV relieved the CSF obstruction caused by the tumor and hemorrhage, obviating the need for a diverting shunt. This aligns with the literature, where ETV is often the first-line treatment for obstructive hydrocephalus in tectal gliomas [11]. However, intratumoral hemorrhage adds complexity, and the risks of treatment, such as re-hemorrhage or worsening intracranial pressure, must be carefully considered [5]. A notable case reported by Khalatbari et al. documented a fatal intratumoral hemorrhage in a patient with a tectal plate glioblastoma multiforme (GBM) following ETV placement [12]. In that case, the hemorrhage worsened the elevated intracranial pressure, eventually leading to the patient’s death. This highlights a key concern in cases where ETV is performed. Although it can effectively relieve obstructive hydrocephalus, it may also exacerbate intratumoral pressure in certain settings, particularly in higher-grade gliomas [12].

Similarly, complications have been described in patients with brainstem gliomas, where the tumors’ vascularity and location predispose them to hemorrhage [13]. It is crucial to recognize that hemorrhage can occur even in low-grade gliomas, as reported in Oka et al.’s case of a hemorrhagic pilocytic astrocytoma in the tectal region [14]. Intratumoral hemorrhage has also been linked to CSF diversion procedures in other types of brain tumors, such as pineal tumors and diffuse midline gliomas. These hemorrhages can significantly worsen intracranial pressure and require rapid intervention [15, 16]. Moreover, prior studies have emphasized the need for careful monitoring after any CSF diversion procedure, especially in cases where the tumor’s vascular nature predisposes it to hemorrhage [5, 17].

Further complicating the picture is the challenge of distinguishing mass effect from tumor growth versus intratumoral hemorrhage on imaging. Previous studies have described how even minor hemorrhagic changes could rapidly evolve, leading to significant neurological deterioration [18]. As such, clinicians must maintain a high index of suspicion when evaluating patients with known tectal gliomas, particularly if new symptoms arise suddenly. Regular imaging and follow-up are essential for early detection and management of complications [7].

Given the rarity of documented cases of intratumoral hemorrhage in tectal gliomas, cases like ours and that of Khalatbari et al. suggest the need for heightened vigilance [12]. Even low-grade tumors can undergo sudden hemorrhagic transformations, and the risks of such events should be considered when managing obstructive hydrocephalus with CSF diversion techniques [13, 14]. Early intervention, including surgical resection in select cases, may be required to prevent further hemorrhagic episodes or intracranial hypertension [19]. ETV remains an effective treatment for obstructive hydrocephalus, but clinicians should be aware of the risks of intratumoral hemorrhage in tectal gliomas. Early recognition, regular imaging, and close monitoring are paramount for ensuring optimal outcomes and mitigating the risk of fatal complications [20].

## Conclusions

In this case report, we present a 43-year-old man who presented with severe obstructive hydrocephalus secondary to intratumoral hemorrhage of a known tectal plate glioma. Initial presentation of obstructive hydrocephalus from intratumoral hemorrhage is exceedingly rare. However, clinicians must be aware of this condition, as it can become rapidly fatal if undetected. ETV is a reasonable and well-tolerated intervention for this presentation, but care must be taken to reduce the risk of hemorrhagic complications. When intratumoral hemorrhage is diagnosed and addressed rapidly, patients can have good outcomes with ETV and recover baseline neurological function.
